# The clinical impact of chronopharmacology on current medicine

**DOI:** 10.1007/s00210-025-03788-7

**Published:** 2025-01-10

**Authors:** Mert Kaşkal, Mustafa Sevim, Gökay Ülker, Caner Keleş, Berna Terzioğlu Bebitoğlu

**Affiliations:** 1 Department of Pharmacology, School of Medicine, Marmara University, Istanbul, Turkey; 2 Department of Physiology, School of Medicine, Marmara University, Istanbul, Turkey

**Keywords:** Chronophysiology, Personalized medicine, Circadian rhythm, Chronopharmacodynamics, Chronopharmacokinetics

## Abstract

One of the goals of clinical pharmacology is to optimize patient treatment by adopting new treatment strategies which will increase the efficacy of the treatment and decrease the adverse effects of the drugs. In the literature, it has shown that the effectiveness and toxicity of medications can vary significantly based on when they are administered, making timing a crucial factor in treatment plans. Chronopharmacology a relatively new branch of clinical pharmacology focuses on adjusting drug administration times to enhance patient outcomes. Chronopharmacology is largely influenced by an individual’s circadian rhythm which refers to periodic changes in biological processes depending on the time of the day. The chronopharmacology influences clinical practice, and the accumulating knowledge in this field will likely lead healthcare providers to adopt new strategies for drug treatment regimens. This review aims to summarize the impact of chronopharmacology particularly on current clinical practices and highlight the latest findings related to chronophysiological mechanisms.

## Introduction

Chronopharmacology is a branch of pharmacology that studies how the timing of drug administration affects the body’s response to medication. It emphasizes the role of biological rhythms, such as the circadian rhythm, in influencing drug effectiveness and safety. The studies on chronopharmacology are divided into two main areas: chronopharmacokinetics and chronopharmacodynamics.

Chronopharmacokinetics investigates how the timing of drug intake impacts the processes of absorption, distribution, metabolism, and elimination of the drugs. Chronopharmacodynamics examines the change in the effects of drugs on cellular and tissue levels, depending on the administration time of the drug. Both of these disciplines form the core of chronopharmacology and are the main themes in the aspects of chronopharmacology. They are essential for guiding the optimal timing of drug treatment to enhance benefits and reduce adverse effects.

From a clinical perspective, the fundamentals of chronopharmacology have also been integrated into the current medicine (Fujimura and Ushijima 2023). Especially in the current clinical practice of cardiology, psychiatry, oncology, and immunology, the principles of chronopharmacology influence the drug treatment regimens and the timing of drug administration in the treatment of various diseases within these specialties (Ayyar and Sukumaran 2021). Melatonin agonists and melatonin compounds used in the treatment of insomnia are administered before bedtime to align the body’s circadian rhythm (Williams et al. 2016). In the treatment of dyslipidemia, some experts suggest administering HMG-CoA reductase inhibitors at night because cholesterol synthesis peaks during the night, ensuring higher concentrations of these drugs when cholesterol synthesis is at its highest (Wallace et al. 2003). Histamine-2 receptor antagonists, which are used in the treatment of gastroesophageal reflux disease and peptic ulcers, are administered at bedtime to prevent nocturnal acid secretion and reduce the risk of complications associated with these conditions (Rackoff et al. 2005). As seen in these examples by aligning drug administration with the body’s natural rhythms, the delivery of drugs can be enhanced, and this minimizes fluctuations in drug levels, potentially improving patient outcomes (Patel et al. 2024). Also in terms of personalized medicine, chronopharmacology has an important role because the difference in the circadian rhythm of the patient can cause alterations and pitfalls in the drug treatment regime of the individual. To optimize therapeutic outcomes and minimize side effects, it is essential to assess each individual’s circadian rhythm and tailor their medication schedule accordingly (Lévi et al. 2024).

In this review, we aimed to define the principles of chronopharmacology together with chronophysiology and the topics that form the foundations of this discipline. Additionally, we focused on evaluating recent developments in chronopharmacology and assessing their impact on current clinical applications.

## Chronophysiology

Nearly all biological processes in the human body are governed by various rhythms, which can be categorized into diurnal and circadian rhythms. Diurnal rhythms depend on external environmental signals, whereas circadian rhythms are independent of these signals. However, in practice, this distinction is often overlooked, as most diurnal rhythms have been shown to be circadian in nature (Vitaterna et al. 2001). Biological rhythms can further be divided into ultradian, circadian, and infradian cycles based on their duration. Ultradian rhythms have cycles shorter than a day, circadian rhythms have cycles of approximately 24 h, and infradian rhythms have cycles longer than 24 h (Coskun et al. 2023).

These rhythms play a crucial role in regulating physiological processes. For example, circulating blood cells exhibit periodic changes influenced by factors like cell distribution, proliferation, and destruction (Ohkura et al. 2007). In the cardiovascular system, heart rate, blood pressure, and substrate metabolism display distinct oscillations throughout the day (Schroder et al. 2013). Additionally, while some molecules like nicotinamide adenine dinucleotide (NAD) and nicotinamide adenine dinucleotide phosphate (NADP) in the blood do not exhibit diurnal variations (Fukuwatari and Shibata 2009), anterior pituitary hormones follow a circadian rhythm that can be influenced by various factors (Beyer et al. 2022).

These biological rhythms enable organisms to adapt to daily environmental changes, optimizing bodily functions accordingly. The circadian timing of drug administration and disease manifestation highlights the significance of these rhythms. For instance, cortisol levels, which act as anti-inflammatory agents, are highest upon awakening and lowest at night (Pulopulos et al. 2020), while histamine levels, which can mediate bronchoconstriction, exhibit nocturnal peaks (Nakamura et al. 2017).

The mechanisms underlying circadian rhythms involve a complex interplay of neural and molecular processes, with the primary clock located in the suprachiasmatic nucleus (SCN) of the hypothalamus. The SCN generates self-sustaining circadian rhythms synchronized by external cues (Abarca et al. 2002). The core molecular mechanism within the SCN is driven by feedback loops among rhythmically expressed clock genes and their protein products. Key clock genes, such as period (Per) and cryptochrome (Cry), regulate the oscillations of circadian rhythms (Perrin et al. 2006, Narasimamurthy et al. 2012).

The circadian clock system imposes rhythms and regulates various physiological processes, including the rest-activity cycle, endocrine system, and metabolism. Disruptions in circadian rhythms, such as those caused by sleep loss or chronic sleep deprivation, can have significant physiological effects (Narasimamurthy et al. 2012). Posttranslational modifications, such as the phosphorylation of clock genes by casein kinase I, regulate key molecular mechanisms in the circadian clock (Agostino et al. 2008).

Moreover, the molecular regulation of circadian rhythms involves transcriptional and translational feedback loops orchestrated by clock genes and their protein products (Huang et al. 2024). MicroRNAs, such as miR-192/194 and miR-455-5p, have been identified as regulators of the period gene family and clock mRNA, influencing circadian rhythms at the molecular level (Nagel et al. 2009, Cheng et al. 2022). The miR-192/194 cluster has been shown to modulate the expression of core circadian genes, particularly by repressing the 3′ untranslated regions (UTRs) of genes such as Per1, Per2, and Per3. This repression leads to a shortening of the circadian period of Bmal1 mRNA rhythms in NIH/3T3 fibroblasts, indicating that miR-192/194 is a significant regulator of circadian timing mechanisms (Nagel et al. 2009). Similarly, in studies involving cells and transgenic mice, it was shown that inhibition of miR-455-5p can enhance the amplitude of synchronized rhythms and shorten the periods (Cheng et al. 2022). Amyloid-beta (Aβ) has been implicated in disrupting circadian rhythms, particularly in the context of neurodegenerative diseases such as Alzheimer’s disease. Aβ-induced alterations in the expression of key clock proteins, including BMAL1 and CBP, have been shown to lead to significant disruptions in circadian rhythms (Schmitt et al. 2017, Song et al. 2015).

Understanding the synchronization of biological rhythms with environmental factors, such as the light-dark cycle and feeding schedules, can enhance the effectiveness of treatments. For instance, the suprachiasmatic nucleus (SCN) in the hypothalamus serves as the master clock, regulating these rhythms and ensuring that bodily functions are synchronized with environmental changes (Douma and Gumz 2018). Disruptions in these rhythms can lead to various health issues, including mood disorders and metabolic syndromes (Bulbul et al. 2020, Lee 2019). Optimizing the timing of drug therapy to align with the circadian rhythm can maximize therapeutic effects while minimizing adverse effects. For example, studies have demonstrated that the pharmacokinetics of certain medications, such as antidepressants, can vary depending on the time of administration relative to the body’s biological clock (Lee 2023, Silva et al. 2021).

## Chronopharmacology

### Chronopharmacokinetics

Circadian changes broadly impact the features which affect the process that drugs undergo once they are applied. The most crucial and significant change is the differing blood flow rates of the relevant organ/system. It should not be neglected that the environmental factors also differ daily so these effects can be unpredictable. Also, there are some functional and transcriptional changes in the organs and regulatory systems. Regarding the pharmacological aspects, patient and medical conditions should be assessed individually, as they differ in the key aspects of pharmacokinetics (Daali 2022, Suwinski et al. 2019). Therefore it is important to get a closer look at the pharmacokinetic changes to avoid adverse effects of the drugs and optimize the efficiency of the treatment procedures (Shah and Shah 2024, Erkekoglu and Baydar 2012).

When drugs are administered orally, they undergo a complex absorption phase that is highly affected by visceral blood flow, gastric emptying, gastrointestinal motility, and gastric pH (Voigt et al. 2019). Another confounding factor which may disrupt the absorption of drugs is the expression rates of transporters such as P-glycoprotein (P-gp), ATP-binding cassette (ABC), breast cancer resistance protein (BCRP), and multidrug resistance pump (MDR). In addition, increased gastrointestinal motility and gastric emptying typically result in higher bioavailability during the daytime, particularly for lipophilic drugs (Dobrek 2021). Another factor which regulates the ionization of the drugs and solubility is the gastric pH which is typically lowest just before midnight. Therefore, alkaline drugs may have reduced bioavailability when administered under these conditions. Studies have shown that these diurnal changes in absorption are not limited to oral routes but are also affected by parenteral administration (Bicker et al. 2020).

Drug distribution also shows diurnal variations. Largely depending on the circulatory changes, higher cardiac output is expected during the day as the distribution of the administered drugs. It is known that some transporters and expression of efflux proteins change diurnally which needs further studies for specific drug and distributed tissue combinations. One study reported that protein expressions of the blood-brain barrier (BBB) have diurnal changes (Ogata et al. 2022). Another study demonstrated variations in brain uptake of donepezil, resulting from differences in ATP-binding cassette superfamily G member 2 (ABCG2) expression (Furtado et al. 2024). The drugs have different binding patterns which can be tissue and protein specific. The total amount and free fraction of these proteins also vary throughout the day. A good example is the plasma protein transcortin which binds to corticosteroids. Additionally, fluctuations in plasma concentrations of certain chemotherapeutic and anti-seizure drugs can result in fewer side effects when administered at specific times of the day (To et al. 2000).

The body’s circadian rhythm affects various aspects of GI function, including motility and blood perfusion. For instance, gastrointestinal motility, which is the ability of the digestive system to move its contents through the GI tract, can vary throughout the day. This variation can impact the time a drug remains in the absorption site and, consequently, the extent of its absorption. Similarly, intestinal blood perfusion, which is the blood flow through the intestinal blood vessels, fluctuates over the day, affecting the drug’s absorption rate. Circadian rhythms also influence the expression of membrane transporters and metabolic enzymes in the intestine (Piedras et al. 2024).

Tissue perfusion, the blood supply to tissues and organs, can vary with circadian rhythms due to changes in physical activity and metabolic rates. Especially during periods of rest, blood flow to certain organs may decrease, while during active periods, it may increase, affecting the amount of drug that reaches specific tissues. This variation can impact the drug’s effectiveness and duration of action (Dobrek 2021).

Circadian rhythms can significantly influence regional blood flow, which affects drug distribution and clearance. During periods of increased cardiac output (daytime for diurnal species), blood flow to specific tissues (e.g., liver, kidneys, brain) may increase, enhancing drug delivery and metabolism (Jones and Rowland-Yeo 2013). Conversely, during rest phases, reduced blood flow could limit drug distribution to these tissues. Tissue clearance can be described as CLtissue = *Q* × *E*, where *Q* is the blood flow to the tissue and *E* is the extraction ratio of the drug by the tissue. The blood flow can be influenced by circadian variations, and the change in the blood flow to the tissue may influence clearance.

To optimize energy regulation, the organism performs changes that regulate drug detoxification just as with the food intake and digestion. The liver and kidneys are the main metabolizers of the body. Besides the differing blood flow rates and plasma protein binding rates, the expression of the enzymes varies with the biological rhythm. Both phase I and II enzymes are affected by the circadian changes. The different and complex behaviors of clock genes (Bmal1, CLOCK) which affect cis-elements (E-box, D-box, Rev-erb) or nuclear receptors (HNF4a, PPARG) lead to cycles of drug-metabolizing enzyme rates throughout the day (Lu et al. 2020). Different factors and enzymes have different patterns. Variations are also seen in cytochrome p450 enzymes, which are the major enzymes responsible for drug metabolism. These variations may result in changes in drug metabolisms (Lynch and Price 2007). Also, the activity of these enzymes is influenced by the circadian rhythm, fluctuating during the day due to their circadian properties. CYP 3A4 which can be defined as the most common cytochrome P450 enzyme is also affected by circadian rhythm. This variation is mostly influenced by the circadian properties of nuclear receptors such as pregnane X receptor (PXR) and constitutive androstane receptor (CAR) which controls the regulation of CYP 3A4 enzyme (Daujat-Chavanieu and Gerbal-Chaloin 2020). CYP 2D6 which is responsible for the metabolism of psychotropic medications and beta-blockers is also thought to be affected by circadian rhythm attributed to the fluctuating levels of drugs metabolized by these enzymes during the day (Lu et al. 2020). Additionally, CYP2E1 and CYP3A11 have a higher expression at daytime, while CYP2B10 is expressed more at night (Zhang et al. 2018). Sulfation reactions, which are among the most common phase II reactions, may be influenced by circadian rhythms, with activity shown to peak at night, whereas other phase II reactions are more prominent during the daytime (Dobrek 2021). This variation’s significance depends on the extraction of the compound. If drug metabolism is rapid, it is more likely to be affected by the blood flow changes in the liver.

Excretion also varies with circadian changes affecting both urine and bile secretion. Hydrophilic drugs are mostly excreted by urine. For renal excretion, the drug excretion rates depend mainly on the glomerular filtration rate (GFR) and renal blood flow. As expected, it has been shown that the GFR is usually high during daytime (Dobrek 2021). While this depends on cardiac output, it remains unclear whether other factors contribute to this phenomenon. Aminoglycosides are famous for their hydrophilic nature and nephrotoxicity. One study showed that gentamicin causes fewer side effects and nephrotoxicity when applied in the morning (Prins et al. 1997). In addition, the pH of the urine is another factor which affects renal excretion. It influences the ionization of the drugs in the urine. The pH of urine is determined by the H+ secretion. In proximal tubules, there is a transporter called sodium-hydrogen antiporter 3 (NHE3). This transporter has a huge role in acid secretion and may be affected by the circadian rhythm (Saifur Rohman et al. 2005). The pH values of the urine are lower in the morning, after the night rest (Bicker et al. 2020). If the urine is acidic, acidic drugs tend to be reabsorbed more efficiently from the urine. In an acidic environment, acidic drugs tend to be reabsorbed more efficiently from the urine, as they are more likely to exist in their non-ionized, lipophilic form.

In conclusion, chronopharmacokinetics is a crucial subject for increasing the efficacy and safety of pharmacological treatment. Although there are numerous contributing factors and it may seem only that drug-based holistic studies are beneficial; understanding the underlying mechanisms is essential in order to adjust for confounding factors and optimize treatment outcomes and modalities.

### Chronopharmacodynamics

In chronopharmacological studies, data has been obtained indicating that despite administering the same dose of a drug, the effect of the drug may vary depending on the time of administration (Ayyar and Sukumaran 2021). These variations in drug efficacy are attributed to changes in receptor availability and the protein structures that generate responses, which may fluctuate depending on the time of the given drug, thereby influencing differences in responses of the same drug doses (Takane et al. 2000).

The administration of interferon β to rodents was found to be more effective when the drug was given at 9.00 am versus 9.00 pm, and this effect may be linked to a change in interferon β receptor sensitivity as well as an increase in interferon β receptor levels due to the change in receptor behavior depending on the time of the day (Mager et al. 2003). Also, the time-dependent increase in efficacy may be caused by the changes in the feedback mechanisms involving secondary proteins. High glucocorticoid concentrations suppress the receptor activity, particularly on epidermal growth factor which was found to be higher in rodents in daytime compared to nighttime (Lauriola et al. 2014). Epidermal growth factor has an effect on the growth and regeneration of tissues as well as cell interactions; therefore, this change in the activity of epidermal growth factor receptors may cause differentiation in homeostasis depending on the time of the day.

The density and responsiveness of the beta-adrenergic receptors follow a circadian pattern with high receptor density and sensitivity during daytime (Lemmer 2007). Beta-receptor antagonists, commonly used as anti-arrhythmic drugs in clinical practice, can be influenced by circadian properties. Sympathetic activity is generally higher during the day, which may lead to increased heart rate and arrhythmias. This suggests that administering beta-receptor antagonists during the early part of the day might be more effective (Wang et al. 2021). Additionally, parasympathetic response which may increase at night could influence bronchoconstriction which will exacerbate the symptoms of asthma and chronic obstructive pulmonary disease. Therefore, administering beta-receptor agonists as bronchodilators at night, when circadian factors intensify these symptoms, could be more effective in controlling them (Fink et al. 2018). Similarly, histamine H1 receptors show diurnal variation, with receptor activity higher at night which may contribute to the exacerbation of allergic symptoms during night (Reinberg 1997). Histamine H1 receptors also play an important role in wakefulness and attention, and the circadian properties of these receptors show that they may be more active during the daytime to maintain arousal (Morioka et al. 2018). Because of the circadian properties of histamine 1 receptors, the histamine receptor antagonist is mainly administered at night to better cope with insomnia and allergic symptoms (Huang et al. 2011). In contrast, insulin receptor sensitivity tends to increase in daytime which leads to increased effects of insulin-mimetic drugs when administered in the early morning (Singh et al. 2017). Opioid receptors which exhibit circadian variation in sensitivity tend to be more responsive in the evening compared to the daytime, suggesting that lower doses of opioid receptor agonists may be required to maintain pain relief when administered in the evening (Boom et al. 2010). However, no definitive effect has been established regarding the optimal timing of opioid treatment for pain relief, and clinical studies are needed to confirm these findings.

The field of chronopharmacodynamics lacks clinical studies to establish a conclusive link between the time of drug administration and clinical outcomes. More comprehensive studies are needed to better understand the circadian dynamics of receptor activity to optimize drug efficacy and minimize adverse effects.

## Clinical effects of chronopharmacology

### Chronopharmacology and cardiovascular medicine

The effect of circadian rhythm on blood pressure can serve as a good example of the relationship between chronopharmacology and cardiovascular medicine. Differences in blood pressure levels can be observed throughout the day, depending on daily activities and changes in the autonomic nervous system. While the blood pressure is typically higher during the day, it decreases to lower levels at night. It has been shown that blood pressure reaches peak levels in the late morning, and it starts to decline at night, especially after 8 pm (Smith 2001). The parasympathetic part of the autonomic nervous system plays a crucial role in the homeostasis of blood pressure in addition to decreased renin-angiotensin activity and the decreased secretion of pressor hormones which leads to a decrease in blood pressure and heart rate at night (Lemmer 2012).

Since blood pressure naturally fluctuates throughout the day, peaking in the morning and decreasing at night, treatment of hypertension can follow chronopharmacological principles. Given the physiology of blood pressure, blood pressure–lowering anti-hypertensive drugs should be given after awakening the patient in the morning when blood pressure is higher (Potucek and Klimas 2020).

It is important to note that hypertensive patients can be classified as either dippers or non-dippers. In dipper-type patients, blood pressure levels follow normal physiological patterns with higher readings in the morning and afternoon followed by a decline at night. In contrast, non-dipper patients experience consistently high blood pressure levels at nighttime, and clinical studies have shown that these patients have a higher risk of cardiovascular complications (Chotruangnapa et al. 2021). For the prevention of cardiovascular complications and the surge in blood pressure levels at nighttime, in non-dipper patients, the anti-hypertensive drugs can be dosed at nighttime in selected non-dipper patients.

The morning period is also recognized as an increased risk factor in cardiovascular events such as acute coronary syndromes and strokes. This increase may be linked to decreased fibrinolytic activity which may lead to an increase in thrombus formation in the vessels and increased sympathetic activation of the autonomic nervous system (Smith 2001). In patients prone to cardiovascular events, the drugs to prevent cardiovascular complications such as beta-receptor blockers may be administered early in the morning. The administration of beta-blockers in the morning also diminishes the effects of the increased sympathomimetic activity which may lead to an increase in cardiovascular complications.

Cholesterol synthesis predominantly occurs at night, a process driven by circadian rhythms. The enzyme HMG-CoA reductase, which is the rate-limiting step in cholesterol synthesis, has increased activity, especially in the evening and nighttime (Le Goff et al. 2001). Due to the heightened cholesterol synthesis at nighttime, the administration of cholesterol-lowering drugs such as statins is more effective at nighttime compared to morning dosing (Awad et al. 2017). Additionally, statins have mild adverse effects such as myalgias and muscle cramps, and the night dosing of these drugs will cause peak concentration in sleep time and minimize discomfort during daily life activities. Especially nighttime dosing is important for statins with shorter half-lives such as simvastatin and fluvastatin to increase the effect of these drugs. However, recent studies showed that the most commonly used statins are rosuvastatin and atorvastatin, which have longer half-lives and could be administered any time in the day without an alteration in cholesterol-lowering effects (Vizdiklar et al. 2024).

### Chronopharmacology and endocrinology

The hormones in the body generally follow a circadian rhythm, and therefore, the timing of medications which affect the endocrine systems may change the efficacy as well as adverse effects of these drugs. Especially studies on thyroid drugs, glucocorticoids, and anti-diabetic drugs are well documented, and the effect of the administration time of these drugs on the disorders of these systems is investigated.

The thyroid hormones, thyroxine (T4) and triiodothyronine (T3), follow a circadian rhythm. Their levels fluctuate through the day, peaking in early evening and morning and declining in the afternoon (Ikegami et al. 2019). For patients who need replacement therapy of thyroid hormones which is mostly levothyroxine, a synthetic form of T4, the medication is usually recommended to be taken approximately 30–60 min before breakfast on an empty stomach, due to delayed drug absorption with food (Ala et al. 2015). However, it is suggested that levothyroxine can be taken at bedtime several hours after the meal (again in a fasting state), this is because the natural peak of thyroid stimulant hormone (TSH) occurs at night, and it is thought that this may enhance the patient response to levothyroxine treatment (Bolk et al. 2010).

Glucocorticoids are closely linked with chronopharmacology, and the side effects and efficacy of glucocorticoid treatment can be significantly affected by the timing of drug administration. Especially cortisol, the primary glucocorticoid in humans, follows a strict circadian rhythm. It peaks around in the early morning and reaches its lowest level around midnight (Nicolaides et al. 2015). This rhythm is controlled by the hypothalamic-pituitary-adrenal (HPA) axis which is influenced by the signals such as light exposure, stress levels, and sleep-wake cycles. Glucocorticoids are generally used in auto-inflammatory diseases, and the administration of glucocorticoids in the morning will reduce the morning stiffness and pain caused by these conditions as well as the side effects of these drugs (Oster et al. 2017). As a result in common clinical practice, these drugs are taken in the early morning.

Circadian rhythms regulate glucose metabolism by affecting pancreatic beta-cell function, insulin sensitivity, and glucose production in the liver (Qian and Scheer 2016). Especially studies suggest that insulin sensitivity tends to be higher in the morning compared to the evening (Saad et al. 2012). It is stated that the evening administration of metformin would have a better effect on the control of blood glucose levels because hepatic glucose production peaks in the evening and night (Chan et al. 2022). Also, in terms of insulin treatment, the administration of insulin late evening and night would maintain a more stable background insulin throughout the day and counteract the dawn phenomenon which is an early morning rise in blood glucose levels due to the circadian rhythm of glucose (Morris et al. 2015).

### Chronopharmacology and neurology

The treatment of neurological disorders is also related to chronopharmacology. The use of chronopharmacological principles in neurological diseases such as epilepsy and migraine may help improve drug efficacy and decrease adverse effects.

Epileptic seizures often occur in the early morning, particularly in cases of generalized epilepsy (Bazil and Walczak 1997). This observation suggests administering anti-epileptic drugs early in the morning to prevent epileptic seizures. However, different types of epileptic seizures may follow a nocturnal pattern and occur more frequently at night (Herman et al. 2001). Based on these patterns, a personalized approach may be more suitable for epilepsy patients to increase the efficacy of the treatment.

Migraine headaches can also show a circadian rhythm, and many headaches are seen more frequently in the morning (Kelman 2007). This occurrence of morning migraines is linked to fluctuations in cortisol and melatonin levels throughout the day (Baksa et al. 2019). The morning surge in cortisol levels is believed to be the primary factor contributing to the increased frequency of migraine attacks in the early morning. Due to this connection and the timing of migraine attacks, some studies suggest that preventive migraine drugs and acute migraine medication should be taken in the early morning to reduce the severity and frequency of migraine headaches (Cady et al. 2004).

Although chronopharmacological approaches have been proposed for the treatment of neurological disorders, no consensus has been established regarding the timing of drug administration for these conditions, and the decision should be tailored to the disease pattern of each individual patient.

### Chronopharmacology and psychiatry

The occurrence of depression can vary due to the effect of seasons on the mood. In winter, especially in Nordic countries, reduced exposure to sunlight and longer nighttime hours may have a negative impact on the mood of people living in these regions. This seasonal effect on mental health is called seasonal affective disorder (SAD), and the patients may require treatment for this condition (Munir et al. 2024). SAD symptoms may include atypical features like hypersomnia, overeating, significant fatigue, and other features of major depressive disorder (Forneris et al. 2019). In this condition, decreased sunlight exposure in autumn and winter can disrupt the homeostasis and circadian rhythm of the body, and this may cause decreased serotonin levels leading to depression (Lam et al. 2006). Also, the change in circadian rhythm due to low sunlight exposure can disrupt the balance of melatonin which is important in balancing the mood and sleep of the individual (Lam and Levitan 2000).

The treatment of SAD typically involves light therapy, where patients sit near a lightbox that mimics natural sunlight for about 20–30 min each morning (Campbell et al. 2017). This treatment can help reset circadian rhythms and alleviate depressive symptoms. Additionally, selective serotonin re-uptake inhibitors (SSRI) may be beneficial in the autumn and winter period and also can be initiated before the beginning of symptoms in early autumn (Modell et al. 2005).

Chronobiology also plays a significant role in sleep patterns. The use of melatonin and drugs that modulate melatonin receptors is important in the treatment of insomnia. Under normal physiological conditions, melatonin levels are low during the daytime and begin to rise in the evening to promote sleep. In the treatment of insomnia, melatonin and melatonin receptor agonists are also given in this manner to mimic the chronophysiological pattern. Melatonin and derivates are typically taken 1 h prior to bedtime to induce and ensure the continuity of sleep (Golombek et al. 2015, Neubauer et al. 2018).

### Chronopharmacology and immunology

The circadian rhythm, governed by internal biological clocks, plays a crucial role in regulating various physiological processes, including immune responses (Dallmann et al. 2014). Understanding the time-of-day-dependent functions of immune cells, such as macrophages, is essential for developing personalized health strategies and advancing chronobiology and chronopharmacology-based treatments for infectious diseases (Shirato and Sato 2022). Daily oscillations in host-microbial interactions have been shown to influence the effectiveness of antimicrobial and anti-inflammatory therapies, underscoring the importance of considering the circadian coordination of immune responses in treatment strategies (Tognini et al. 2017).

The circadian clock regulates immune functions by orchestrating rhythmic processes within the immune system. This regulation optimizes immune surveillance, responsiveness, and homeostasis, thereby enhancing resilience against diseases (Ding et al. 2024). The circadian clock influences the temporal regulation of cytokines, gene expression, and immune cell activities, driving the daily rhythms observed in immune responses (Nakao 2014). At the molecular level, the circadian clock coordinates immune responses by modulating gene expression feedback loops and transcriptional regulation within immune cells (Scheiermann et al. 2013). The circadian system rhythmically shifts the immune system between heightened immunity for optimal pathogen surveillance and reactivity and reduced immune responsiveness to facilitate tissue repair and regeneration (Fonken et al. 2016).

Moreover, the circadian clock influences the expression of immune-related factors such as granzyme B, perforin, IFN-γ, and NK cell cytolytic activity in a rhythmic manner, affecting immune cell functions and cytotoxicity (Arjona et al. 2004, Arjona and Sarkar 2005).

The bidirectional interactions between the circadian and immune systems involve immune factors influencing circadian timing and the circadian system regulating immune functions (Coiffard et al. 2021). Disruption of the circadian system has been linked to inflammatory pathologies, cancer, metabolic disorders, and premature aging, underscoring the critical role of circadian rhythms in immune regulation and overall health (Zhang et al. 2021). Recent research has highlighted the importance of circadian rhythms and sleep in maintaining immune system homeostasis, emphasizing the impact of proper timing on immune responses (Haspel et al. 2020).

### Chronopharmacology and pain management

Pain perception in the body may change depending on the chronobiological rhythm (Junker and Wirz 2010). The timing of analgesic drug administration has been investigated for some time, and it has been reviewed that the efficacy and adverse effects of analgesic drugs may vary depending on the administration time (Ursini et al. 2021). Also, the levels of pro-inflammatory cytokines could change over a 24-hour period (Labrecque and Cermakian 2015). These changes in pro-inflammatory cytokines also promote the differentiation in pain perception during the day. These aspects of chronotherapy concepts have introduced modified released therapies for the control of pain in patients.

In the case of nonsteroid anti-inflammatory drugs (NSAIDs), it has been shown that the morning administration of diclofenac, indomethacin, and ketoprofen showed an increase in the ‘‘C max’’ value by 32%, 52%, and 50%, respectively (Baraldo 2008). This enhancement in the serum levels of these drugs may prove that morning dosing could be more beneficial for the control of pain perception in patients.

In the chronopathology of rheumatoid arthritis, morning serum levels of cytokines, particularly interleukin-6, increase (Petrovsky and Harrison 1998). This rise in cytokine levels is linked to morning stiffness which may heighten pain perception in these patients. Therefore, administering anti-inflammatory drugs in the morning may help alleviate the symptoms of rheumatoid arthritis.

In the management of chronic pain especially with the features of a neuropathic nature, the symptoms tend to worsen at night (Zhu and Huang 2023). This highlights the need for additional analgesic treatment at night, particularly for the pain with neuropathic origin. This situation is especially important in elderly patients with neuropathic pain, as inadequate pain control may cause deprivation in sleep patterns and further complications in these patient groups.

### Chronopharmacology and oncology

The drugs used in oncology are considered highly effective due to their antineoplastic effect but they also exhibit a broad range of severe adverse effects. The adverse effect profiles of antineoplastic agents are an important topic of discussion, particularly in the context of chronopharmacology. The timing of drug administration has been shown to potentially influence both the efficacy and the side effects of these agents. By aligning the administration of antineoplastic drugs with the body’s circadian rhythms, it may be possible to enhance their therapeutic effects while minimizing adverse reactions. Ongoing research is focused on identifying the optimal timing for these treatments, with the aim of improving patient outcomes and reducing the burden of side effects.

5-Floururacil (5-FU) is commonly used for the treatment of colorectal cancer. One of the most common adverse effects seen with the use of 5-FU is mucositis (Baydar et al. 2005). Studies have shown that the administration of 5-FU in the afternoon may cause less mucositis compared to administration in the morning (Giacchetti et al. 2006). This effect is likely due to the circadian activity of the enzyme dihydropirimidine dehydrogenase (DPD) which metabolizes 5-FU, with lower activity in the evening (Ballesta et al. 2017). Oxaliplatin, often given in combination with 5-FU, especially for colorectal cancer treatment, has been shown in some studies to cause less nephrotoxicity when administered at night, without reducing its efficacy (Innominato et al. 2014). This may be related to the circadian rhythm of DNA repair mechanisms, which are more active during the day, allowing for better repair of oxaliplatin-induced DNA damage in healthy cells, potentially reducing side effects (Sancar et al. 2015). Cisplatin is also used in various cancers including lung and ovarian cancers and has also been suggested to cause less nephrotoxicity when administered in the early morning (Dallmann et al. 2014). This is due to the circadian rhythm of the kidney where repair mechanisms are more active in the morning thereby reducing the drug-induced nephrotoxicity (Odaet al. 2014).

The level of melatonin is also important regarding its role in oncology. Melatonin is one of the primary hormones that follows a circadian rhythm and is commonly used in the treatment of insomnia (Hill et al. 2015). Lower melatonin levels have been linked to cancers, particularly breast cancer (Talib et al. 2021). The link between breast cancer and melatonin may be due to the effect of melatonin on estrogen receptors, and it was shown that melatonin can inhibit the production of estrogen and reduce the expression of estrogen receptors in cells which may inhibit the development of breast cancer (González-González et al. 2018). The International Agency for Research on Cancer also classified working in nightshift as a situation which may alter melatonin levels, due to its effect on melatonin levels (Zhang and Papantoniou 2019). In in vitro studies and animal models, melatonin has been shown to suppress and inhibit the growth of these cells (Reiter et al. 2017). These findings suggest that melatonin may suppress oncogenic cells, which raises the idea of using melatonin as an adjunctive treatment in cancer therapy. However, more clinical research is needed to better understand melatonin’s role in oncology.

Currently, there are no comprehensive guidelines for determining the optimal timing of oncological drug administration. However, research emphasizes that drug administration timing may be important in cancer patients, and more detailed clinical studies are needed to clarify the role of chronopharmacology in cancer treatment. Ongoing research continues to explore the timing of other chemotherapeutic agents and combinations, as well as the role of chronotherapy in other types of cancer. The goal is to develop more personalized and effective treatment regimens that consider not only the type of cancer but also the optimal timing of treatment administration which could reduce adverse effects and improve treatment efficacy.

## Discussion

Chronopharmacology is an emerging field that explores the relationship between the timing of drug administration and the body’s biological rhythms, particularly circadian rhythms. This discipline is gaining increasing recognition for its potential to enhance therapeutic outcomes and minimize adverse effects in particular areas.

Recent advancements in chronopharmacology have highlighted the significance of aligning drug administration schedules with the patient’s circadian rhythms. This approach has been shown to improve the efficacy of treatments while reducing side effects.

**Fig. 1:**
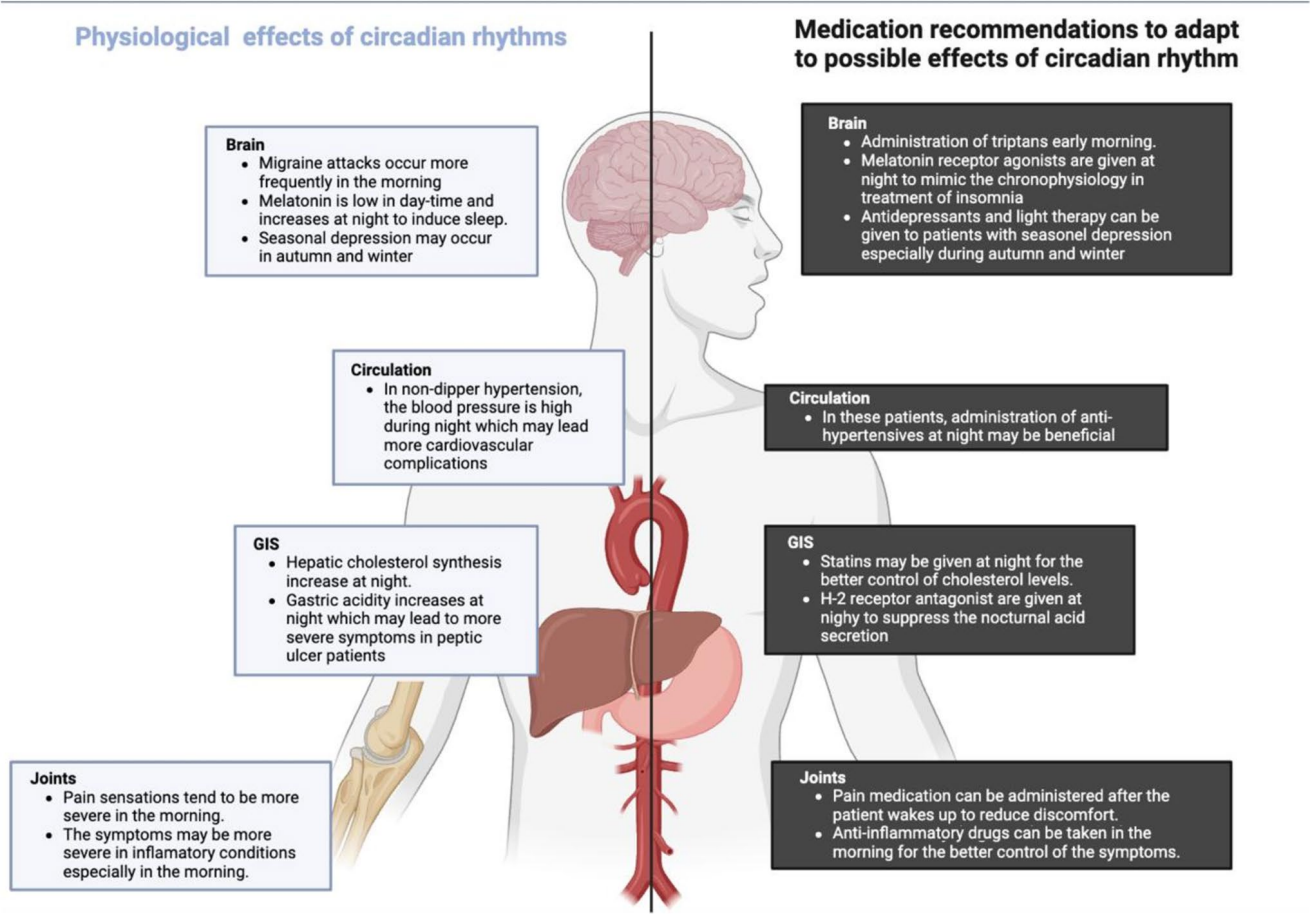
Physiological effects and medication to adapt the possible effects of circadian rhythm. Created in BioRender. Sevim, M. (2024) https://BioRender.com/y55p401

Table 1 outlines the relationship between the timing of drug administration and the management of various health conditions, highlighting the importance of chronopharmacology in optimizing treatment outcomes. Figure 1 shows the physiological effects and possible medication strategies to adapt to circadian rhythms.

**Table 1:** Chronopharmacological recommendations for certain conditions

Condition	Chronopharmacological recommendations
Migraine	Administration of triptans early in the morning, because migraine attacks tend to occur more frequently during this time.
Pain medication	Pain sensation can be more severe in the morning so it is advised to take pain medication in the morning.
Insomnia	Administration of melatonin before going to bed for better management of sleep disturbances
Inflammatory conditions	The symptoms are usually more severe in the morning. It is advised to take non-steroid anti-inflammatory drugs and cortisol after waking up before the daily activities begin.
Peptic ulcer	Histamine-2 receptor antagonists are more beneficial when taken at night, as this helps suppress nocturnal acid secretion.
Hypertension	In non-dipper hypertension patients, the blood pressure does not decrease at night, increasing the risk of complications. These patients may take anti-hypertensive medication at night to prevent complications.
Dislipidemia	Hepatic cholesterol synthesis tends to increase at night due to this the intake of cholesterol drugs at night might be beneficial.
Epilepsy	There might be an increased risk of epileptic seizures in the early morning, so it is advised to take epilepsy drugs after waking up.
Depression	Seasonal depression often occurs in autumn and winter, anti-depressants combined with phototherapy may be prescribed during these seasons.

The timing of anti-hypertensive medication, for example, can influence blood pressure control throughout the day. Studies suggest that administering these drugs at night might provide better blood pressure regulation and reduce the risk of cardiovascular events in some patients (Potucek and Klimas 2020).

Cancer treatments, including chemotherapy, are increasingly being scheduled based on circadian rhythms to maximize their effectiveness and minimize toxicity. Tumor cells and healthy cells may exhibit different sensitivities to drugs at various times of the day, making chronotherapy a promising approach in cancer care.

The effectiveness of psychiatric medications, such as antidepressants and antipsychotics, can be influenced by the time of administration and seasonal changes. Chronopharmacology has the potential to optimize treatment regimens for mental health disorders, improving patient outcomes (Modell et al. 2005).

The immune system’s function is modulated by circadian rhythms, and the timing of immunosuppressive or immunostimulatory treatments can significantly impact their effectiveness. These rhythms influence the activity of immune cells, the production of cytokines, and the overall immune response. The production and release of cytokines vary throughout the day. For instance, pro-inflammatory cytokines like TNF-α and IL-6 tend to peak during the night and early morning hours (Petrovsky and Harrison 1998). This fluctuation can influence the severity of inflammatory diseases and the timing of symptoms, such as increased pain or fever in the morning. Different types of immune cells, including T cells, B cells, and natural killer (NK) cells, exhibit circadian variation in their activity levels (Nobis et al. 2016). This circadian regulation can affect how the body responds to infections, vaccines, and immunotherapies.

For patients with autoimmune diseases or those undergoing organ transplantation, the timing of immunosuppressive drugs can be crucial. Administering these drugs at times when the immune system is less active (e.g., late evening or night) can enhance their efficacy and reduce the risk of rejection or flare-ups.

The circadian rhythm also affects the proliferation of cancer cells and the immune system’s ability to target them. Chronopharmacology can guide the timing of cancer immunotherapies, such as checkpoint inhibitors, to align with periods of peak immune activity, potentially improving treatment outcomes.

In conditions like rheumatoid arthritis, where symptoms are often worse in the morning, timing anti-inflammatory drugs to counteract the early-morning rise in pro-inflammatory cytokines can provide better symptom control (Labrecque and Cermakian 2015).

Recent developments in chronopharmacology have led to the identification of small-molecule modulators that target circadian clock components. These modulators can alter the timing of immune responses and are being explored as potential treatments for inflammatory and autoimmune diseases. Understanding the molecular mechanisms that link the circadian clock to immune function could lead to more precise and effective therapies.

Chronopharmacology also plays a crucial role in personalized medicine by considering individual variations in circadian rhythms. The field recognizes that each patient’s internal clock is unique, and this can lead to variations in how they metabolize and respond to medications.

In conclusion, chronopharmacology represents a significant step forward in the quest to optimize drug therapies. By integrating the principles of this discipline into clinical practice, healthcare providers can enhance the effectiveness of treatments, reduce adverse effects, and move closer to the goals of personalized medicine. Ongoing research and clinical trials will continue to refine these strategies, ultimately leading to more precise and effective treatment protocols across a wide range of medical fields.

## Data Availability

All source data for this work (or generated in this study) are available upon reasonable request.
